# The Use of Fecal Microbiome Transplant in Treating Human Diseases: Too Early for Poop?

**DOI:** 10.3389/fmicb.2021.519836

**Published:** 2021-05-13

**Authors:** Hooi-Leng Ser, Vengadesh Letchumanan, Bey-Hing Goh, Sunny Hei Wong, Learn-Han Lee

**Affiliations:** ^1^Novel Bacteria and Drug Discovery Research Group, Microbiome and Bioresource Research Strength, Jeffrey Cheah School of Medicine and Health Sciences, Monash University Malaysia, Bandar Sunway, Malaysia; ^2^Biofunctional Molecule Exploratory Research Group, School of Pharmacy, Monash University Malaysia, Bandar Sunway, Malaysia; ^3^College of Pharmaceutical Sciences, Zhejiang University, Hangzhou, China; ^4^Department of Medicine and Therapeutics, Li Ka Shing Institute of Health Sciences, The Chinese University of Hong Kong, Sha Tin, Hong Kong

**Keywords:** microbiome transplant, poop, microbiome, FMT, fecal

## Abstract

Fecal microbiome transplant (FMT) has gained popularity over the past few years, given its success in treating several gastrointestinal diseases. At the same time, microbial populations in the gut have been shown to have more physiological effects than we expected as “habitants” of the gut. The imbalance in the gut microbiome or dysbiosis, particularly when there are excessive harmful pathogens, can trigger not just infections but can also result in the development of common diseases, such as cancer and cardiometabolic diseases. By using FMT technology, the dysbiosis of the gut microbiome in patients can be resolved by administering fecal materials from a healthy donor. The current review summarizes the history and current uses of FMT before suggesting potential ideas for its high-quality application in clinical settings.

## Introduction

The Food and Drug Administration (FDA) in the United States (US) recently issued several warnings on fecal microbiome transplant (FMT) and has even planned to halt several FMT clinical trials following numerous infections and one death after FMT procedure (US Food and Drug Administration, 2019a, b, 2020a). FMT is a procedure that delivers specially prepared stool material from healthy donors to the patient (i.e., as a recipient) to improve certain medical conditions by restoring the balance of the gut microbial community. In other words, FMT involves transferring a healthy donor’s “poop” to a patient. It may sound disgusting as the word “poop” or “feces” is generally known as waste or bodily excretion. This perception is not entirely wrong, as the main component of feces would be “carcass” of food after they have passed through our digestive system, with most of their nutrients (presumably) having been absorbed. Nonetheless, in clinical settings, stool samples are often collected for health screening purposes to detect any presence of harmful pathogens or inflammatory status. Given that normal stool (typically brown and a soft to firm consistency) indicates good health, this prompts the question: Can stool be used as a “tool” to improve general health in humans?

**FIGURE 1:**
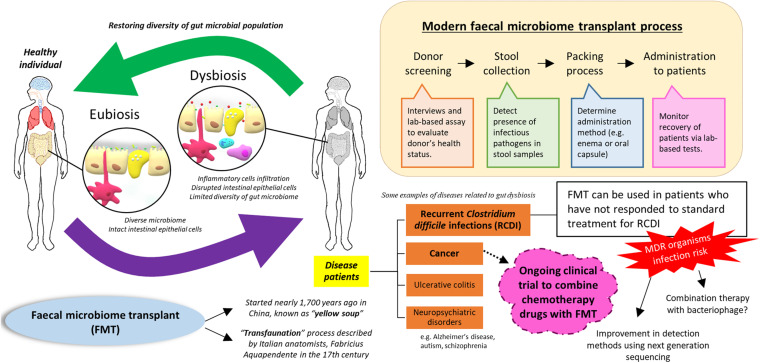
Fecal microbiome transplant use to restore balance in the gut microbiome.

Firstly, the term “gut eubiosis” is widely used to describe aharmonic interbacterial condition, marked by an optimal balance of the microbiome in the digestive tract, as well as a mutualistic relationship between the microbiome and the host that is favorable for human health (Figure 1). Theoretically, microorganisms in our gut would try to compete with incoming microbes (e.g., from food or external contact/environment) in the conquest to secure their habitat and improve their propagation, which ultimately ensures their survival in the human body. However, the gut microbiome would usually be in the state of eubiosis in a healthy individual, carrying a diverse array of microorganisms with considerable tolerance to commensal or “friendly” bacteria (Thaiss et al., 2016; Yang et al., 2016; Lau et al., 2021). In the early 1970s, several American microbiologists had run a few statistical analyses with a series of assumptions and subsequently suggested the ratio of bacteria to human cells to be 10:1 (Luckey, 1970). However, recent studies disapproved this ratio and revised the estimation for the number of human and bacteria cells in the body (Sender et al., 2016). The research group explained that an average human body (with a bodyweight of 70 kg) is made of approximately 200 types of cells and sums up to a whopping total of 30 trillion cells. On the other hand, the mass of bacteria cells in our body is estimated to be 0.2 kg; this figure may not seem to be fascinating, but when you take a deeper look at the bacterial cell count, 0.2 kg of body weight is comprised of 30 trillion bacterial cells! With these findings, Sender et al. (2016) has busted the myth that bacterial cells outnumbered human cells in the body and proposed a revised estimated ratio of bacterial to human cells to be 1:1, with the vast majority of them residing in the colon (approximately 10 trillion cells, 33% of the total bacterial cells). In fact, many studies have examined the development of the gut microbiome in infants; the gut microbiome of infants can differ based on delivery methods (i.e., vaginal birth or cesarean birth), and the cessation of breastfeeding can induce a shift in microbiome towards an adult-like composition (Backhed et al., 2015; Yang et al., 2016; Mohammadkhah et al., 2018). Consequently, it is totally reasonable to acknowledge that the gut microbiome represents a highly dynamic environment in which changes can be induced through diet, medication intake, or even emotional stress (Voreades et al., 2014; Ma et al., 2018; Yu et al., 2018; Lee S.H. et al., 2020; Morrison et al., 2020; Vila et al., 2020).

## Importance of Gut Microbiome in Diseases

In a healthy state, the gut microbiome is predominantly colonized by *Bacteroidetes*, *Firmicutes*, *Actinobacteria*, *Proteobacteria*, and *Verrucomicrobia* bacterial phyla (Jiménez-Avalos et al., 2021). Having that said, the composition and colonization pattern differs slightly between different regions of the digestive tract due to many factors such as pH, oxygen levels, as well as the host immune system (Donaldson et al., 2016; Jiménez-Avalos et al., 2021). Gut dysbiosis occurs when there is a perturbation to the complex commensal microbial communities in the digestive tract, including the overgrowth of certain microorganisms (e.g., bacteria or fungal). As a matter of fact, the risk factors that result in gut dysbiosis are yet to be fully discovered and understood. In infants, apart from delivery methods, some researchers have described the maternal use of antibiotics during pregnancy and labor (i.e., intrapartum) can also influence the infant’s microbiome (Mshvildadze et al., 2010; Gonzalez-Perez et al., 2016; Dunn et al., 2017; Miyoshi et al., 2017; Neuman et al., 2018; Lee J.K.F. et al., 2020; Mitchell et al., 2020; Hoang et al., 2021). Mshvildadze and the team have explored the microbiome of preterm infants and revealed that infants of those who received antibiotics intrapartum have less microbiome diversity than those who did not receive antibiotics (Mshvildadze et al., 2010). This observation is relatively simple to comprehend as antibiotics intake would disrupt the balance in the normal gut microbiome, even though it was supposed to be preventing infection by taking out pathogenic bacteria (Yoon and Yoon, 2018). Moreover, antibiotics use during intrapartum have been shown to be associated with overall microbial composition in breast milk including decreased levels of protective bacteria like *Bifidobacteria*, which then led to the concern that infant would benefit less from drinking the mother’s breast milk and place them at a higher risk of having gut dysbiosis (Bezirtzoglou et al., 2011; Walker, 2013; Gardner et al., 2017; Stearns et al., 2017; Leppälehto et al., 2018; Hermansson et al., 2019; Padilha et al., 2019). Similar observations were also seen in human adults, whereby medication use is often associated with altered gut microbiome and dysbiosis (Francino, 2015; Yoon and Yoon, 2018). For instance, Dethlefsen and team have harnessed the potential of advanced sequencing technology in exploring gut microbiome composition after the use of ciprofloxacin (an antibiotic that is commonly prescribed to treat bacterial infections in the urinary tract, respiratory tract, and so on) instead of traditional culture methods that reveal reduced portion of bacteria growth on media plates (Dethlefsen et al., 2008). Based on their results, the use of ciprofloxacin reduced the diversity and species richness of the gut microbiome, affecting about 30% of bacterial taxa abundance in the gut. More importantly, even after some microorganisms did “make a comeback” by 4 weeks after stopping the antibiotic, some failed to recover at a long period of 6 months. In short, the excessive use of antibiotics can alter the community structure of the microbiome, leading to gut dysbiosis. In the long run, misuse of antibiotics fosters antibiotic resistance, resulting in a bigger problem – the emergence of multidrug-resistant microorganisms (Francino, 2015; Yallapragada et al., 2015; Frohlich et al., 2016).

The significance of the gut microbiome in human health emerged as early as 400 B.C. when the Greek physician Hippocrates said, “death is in the bowels” and also “poor digestion is the origin of all evil” (Iebba et al., 2016). Over the years, researchers have discovered that there are strong correlations between the gut microbiome and important systems in the human body, such as the brain in the nervous system and the immune system that protects the body against deadly infections and cancer (Schwabe and Jobin, 2013; Gonzalez-Perez and Lamousé-Smith, 2017; Yang and Jobin, 2017; Chew et al., 2020; Johnson et al., 2020; Lee et al., 2021). Existing evidence has demonstrated that gut dysbiosis can contribute to the etiology of numerous human diseases, including diabetes, atherosclerosis, inflammatory bowel disease, atopic dermatitis, autism, or even the development of cancer (Adlerberth et al., 2007; Michail et al., 2012; Morgan et al., 2012; Mulle et al., 2013; Vinje et al., 2014; Zackular et al., 2014; Song et al., 2016; Gózd-Barszczewska et al., 2017; Jie et al., 2017; Ni et al., 2017; Lee et al., 2020a, b; Selvaraj et al., 2020; Wang et al., 2021). The process of how gut dysbiosis and certain microbial metabolites can lead to mucosal leakiness in the gut and promote inflammation milieu in the entire body by activating specific immune cells has been actively discussed over the past 10 years (Chassaing et al., 2012; Nicholson et al., 2012; Tremaroli and Bäckhed, 2012; Chang et al., 2014; Joyce and Gahan, 2014; Lee and Hase, 2014; Vazquez-Castellanos et al., 2015; Marchesi et al., 2016; Rooks and Garrett, 2016). Using the study by Gózd-Barszczewska and the team as an example to showcase the association of gut microbiome and cardiovascular diseases, they published their results a few years back, noting that individuals with improper levels of total cholesterol displayed *Prevotella*-enriched microbiome (*p* = 0.03) with a decreased abundance of *Clostridium* (*p* = 0.02) compared to those with no cardiovascular disease (Gózd-Barszczewska et al., 2017). Even though *Prevotella* spp. are natural inhabitants in the gut, some of them are now considered as pathobionts and linked with chronic inflammatory conditions (Ley, 2016). For instance, *Prevotella copri*, as one of the “enterotypes” consistently detected in the gut microbiome, is frequently implicated in prompting metabolic changes and enhancing host susceptibility to gut mucosal inflammation (Iljazovic et al., 2020; Wang et al., 2021). By manipulating the gut microbiome through diet, Wang and the team advocated the idea of using precision nutrition to restore the balance in the gut microbiome and consequently reducing the risk of cardiometabolic diseases (Wang et al., 2021). In the same study, the team revealed a strong protective association between Mediterranean-style diet and cardiometabolic disease risk among a subgroup of the participants could be attributed to the absence of *P. copri* in their gut microbiomes. So now, given that the composition of the gut microbiome significantly correlated with human diseases, here comes the question again: How do researchers exploit the fecal microbiome to improve human health?

## The Exploitation of Gut Microbiome and Fecal Microbiome Transplant

The idea of FMT can be traced back to the Chinese civilization, nearly 1,700 years ago in the 4th century when a well-known traditional Chinese medicine doctor named Ge Hong successfully treated patients who had food poisoning and/or severe diarrhea with a human fecal suspension called “yellow soup” by mouth (Zhang et al., 2012; Shi and Yang, 2018). In point of fact, this is not the only record that marks the use of fecal material in Chinese history. Another traditional Chinese medicine practitioner, Li Shizhen, has recorded the use of fecal material to treat abdominal diseases in the most-known book of traditional Chinese medicine known as “Ben Cao Gang Mu” or *Compendium of Materia Medica*. In the West, FMT studies were led by the Italian anatomists named Fabricius Aquapendente in the 17th century; he named the process “transfaunation,” where viable enteric bacteria transplanted from the healthy animal has effectively restored sick animals to health and vigor (Borody et al., 2004; Dodin and Katz, 2014). However, the transfer of fecal material was done differently in humans compared to those of Chinese practitioners. In 1958, the Chief of Surgery at Denver General Hospital, Dr. Ben Eiseman, and his colleagues shared their experience when they productively used fecal enemas to treat four patients reported to have pseudomembranous colitis (Eiseman et al., 1958). It wasn’t until 20 years later, in 1978, when researchers realized that the condition they were treating, pseudomembranous colitis, was likely to be caused by a pathogenic bacterium known as *Clostridioides difficile* (George et al., 1978). It was clearly indicating that these observations did not happen by chance, but some changes must have occurred after the transplant process, regardless of the administrative route. Thinking of it from another angle, what these practitioners and modern researchers have in mind is that through the fecal transplant process, the gut microbiome of the recipient will be forced to go through a “re-education” process and reinstating the balance as the specially treated fecal material can possibly contain more than a thousand functional bacterial species (Zhang et al., 2012; Brandt and Aroniadis, 2013; Smits et al., 2013; de Groot et al., 2017). Indeed, with the advanced sequencing technologies that we have, researchers can now monitor population changes in the microbiome, bypassing the need for cultivation on plates. What’s more interesting is that it was revealed that cross-species FMT might work as well; German soldiers from Afrika Corps had to follow what the natives did and consumed camel stool during World War II, which expedited their recovery from bacillary dysentery when no antibiotics were available (Lewin, 2001). Even though no one was able to explain such a “treatment” method at the time, scientists are now witnessing major progress in science, particularly in the drug discovery sector, using a stool to rehabilitate the gut microbiome of patients (Brandt, 2012; Xu et al., 2015; Gaines and Alverdy, 2017).

Whilst drug in pill form does not sound foreign to many of us, what about pills carrying stool within it? Two years ago, researchers from Canada and China had published their findings from a randomized clinical trial comparing the efficacy of oral capsule containing stool material and colonoscopy route in treating recurrent *C. difficile* infection (Kao et al., 2017). *C. difficile* is classified as a Gram-positive bacteria that is a frequent cause of infectious colitis; this infection is deemed as a complication of antibiotic therapy (Kelly and LaMont, 2008; Honda and Dubberke, 2009; Chai and Lee, 2018). On top of that, recurrent infections are frequently seen in patients; more often than not, it is extremely hard to treat recurrent infections due to its antibiotics resistance, which further justified itself as a high mortality cause in the United States (Honda and Dubberke, 2009; Lessa et al., 2015; McDonald et al., 2018). Based on the findings by Kao et al. (2017), 96.2% of the participants suffering from recurrent *C. difficile* infections were cured of the infections for over 12 weeks after taking the “poop pill,” making it not too different from those received FMT through colonoscopy (with a non-inferiority margin of 15%). Additionally, another team led by Hirsch and Honig in the United States have also observed similar results in recurrent *C. difficile* infections patients treated with specially packed “poop pill” containing feces from healthy volunteers, further substantiating the use of FMT for frail patients (Hirsch et al., 2015). A systematic review was also performed to evaluate the use of FMT to treat *C. difficile* infections, concluding that the administration of fecal material from healthy donors to patients with recurrent *C. difficile* infections is more effective than vancomycin (Moayyedi et al., 2017).

Besides using FMT in treating *C. difficile* infections, some pharmaceutical companies have started trials to examine the potential use of FMT in conjunction with current cancer drugs to ensure higher treatment efficacy (Ryan, 2019). In reality, Stephanie Culler, the co-founder of Persephone Biome, shared her views in a Tedx Talk and started the “Poop for the Cure” campaign to collect feces for cancer therapeutics design (Persephone Biosciences, (n.d)). Within 18 months, the company managed to collect around 1,400 samples around the United States and identified some missing microbes from those who did not respond well to cancer treatment (Ryan, 2019; Culler et al., 2020). The journey on how researchers identify the role of the microbiome in cancer therapy begun when mice with tumors but no gut microbiome showed a different response when treated with drugs like cyclophosphamide, oxaliplatin, cisplatin, and even anti-programmed cell death 1 protein (PD-1) immunotherapy (Iida et al., 2013; Viaud et al., 2013; Gopalakrishnan et al., 2018; Panebianco et al., 2018).

In other words, the gut microbiome plays a significant role in drug metabolism, which then may influence the drug efficacy. This important fact was indeed raised in a recent paper published in *Science* by Rekdal et al. that described gut microbes like *Enterococcus faecalis* and *Eggerthella lenta* can sequentially metabolize levodopa (Ash, 2019; Maini Rekdal et al., 2019). With these findings, it was then described that gut microbes should be taken into considerations when developing new drugs, and perhaps cancer patients could benefit more in terms of recovery by using a “combinatory therapy” plan that involves FMT and certain drugs to restore the balance in the microbiome, while eradicating cancer cells simultaneously.

## Current Problem of FMT: Detection of Potentially Infectious Bacteria

Even though the potential use of FMT therapy in clinical settings has been encouraged in the past few years, that changed from investigational new drug status in 2013 to biologic agent and drug in 2014, FDA abruptly announced on June 13, 2019, that this therapy should be proceeded with cautions following a death of a patient after undergoing FMT and another suffered an invasive infection (US Food and Drug Administration, 2013, 2019a,2019b,2020a; Moore et al., 2014; Woodworth et al., 2017; Allegretti et al., 2019; Kaako et al., 2019). Back in 2019, after examining all possible reasons, these two immunocompromised individuals were found to have received fecal material from the same donor, which contained extended-spectrum beta-lactamase (ESBL)-producing *Escherichia coli*. What was shocking to the community is that the stool samples used were not screened for ESBL-producing gram-negative organisms before use; these findings came to light only after conducting investigations with the stored preparations of FMT from this stool donor, which took place after the occurrence of serious adverse effects. As a result, the FDA has issued a statement that requires clinicians to perform in-depth screening for ESBL pathogens before FMT.

Presently, there are non-profit organizations promoting FMT research among clinicians and scientists (Chen et al., 2020; McCune et al., 2020; Openbiome, n.d.). Like a blood bank that collects blood, this organization works like a “poop” bank that collects feces samples from donors around the United States, and to date, they have obtained more than 3.5 tons of stool and shipped stool materials to seven countries. Due to the recent incidence, the organization has also promoted the importance of “quality check” before FMT procedures, which begins from the first engagement with the donor where he/she is required to undergo a rigorous screening process involving 200 questions and clinical evaluation by internal medicine specialists and a series of biological assays before qualifying as a donor. To ensure the highest quality of stool samples used in FMT, stools are collected continuously from the donor for 60 days, and these materials are kept in quarantine until all assessments (e.g., clinical assessment, stool, and serological testing) are completed. During this period, lab technicians will perform mostly culture-based biochemical assays on each stool sample collected from the donor, coupled with high-throughput sequencing of a specific gene that acts as a “fingerprint” of every microbe, the 16S rRNA gene sequence. Nonetheless, FDA had issued another notice earlier this year to inform two patients developed enteropathogenic *E. coli* (EPEC) infection and four patients developed shiga toxin-producing *E. coli* (STEC) infection after receiving FMT products (US Food and Drug Administration, 2020a). After that, the FDA initiated another round of discussion with OpenBiome where they reached a consensus to implement stricter rules, including additional testing with nucleic acid amplification tests (NAATs) to detect EPEC and STEC (US Food and Drug Administration, 2020c). In addition to that, the organization assured that all the stool samples would be screened according to the new standards to ensure safe access to FMT.

With the availability of next-generation sequencing (NGS) tools like MiSeq and HiSeq systems by Illumina and single-molecule, real-time sequencing (SMRT) technology by Pacific Biosystems, the cost of sequencing has dropped drastically over the past decades (Wetterstrand, 2013; Besser et al., 2018; Solares et al., 2018). Compared to the traditional Sanger sequencing, scientists can now obtain a large amount of genetic information from once-thought problematic samples like stool and identify microbial populations without the need for cultivation. As a screening method, NGS can provide an overview of the microbial population in the stool sample within a short period, and this information can be used to predict the risk of pathogenic infections if used in the FMT process (Besser et al., 2018). Even though the donor may appear healthy, some microorganisms can persist in the body without causing serious infections (Gopinath et al., 2012; Galdys et al., 2014; Zhang et al., 2015). Through NGS, it may potentially improve the outcome of FMT, particularly those who are immunocompromised. Compared to nucleic acid amplification tests (NAATs) that detect specific genes of certain microbes, NGS offers a more rapid solution with a higher depth of information in terms of the microbial population in the samples. Nevertheless, NAATs can be used in combination with NGS; NGS provides an overview of the microbial population during the first screening to ensure the “eubiosis” status of the microbiome, and those who passed the “quality check” can be monitored using NAATs to observe the growth of certain “unwanted” populations.

## Discussion

Even though the skin is thought to be the largest organ in the body, the largest amount of microorganisms is in the gastrointestinal tract instead (Sender et al., 2016). The gut microbiome consists of a mixture of microbes that can be beneficial (i.e., commensal bacteria), and a relatively small portion of them may pose as a disease risk to us (Thursby and Juge, 2017; Bresalier and Chapkin, 2020). Apart from infections, any changes in the equilibrium in the microbial population in the gut have shown to cause or associate with the development of diseases like inflammatory bowel diseases, neuropsychiatric-related diseases, or even cancer (Lee et al., 2019; Johnson et al., 2020; Ternes et al., 2020; Lee and Chang, 2021). As much as gut microbiome composition is affected by diet, recent data have also pointed out that microbes in the gut have functional importance in nutrients and also drug metabolisms (Hooper et al., 2002; Ash, 2019; Maini Rekdal et al., 2019). The entire process may be more complicated than we thought: before considering host-microbial interactions, interspecies microbial interactions can convert pro-drug to drug, or even facilitate the breakdown of an active drug into something useless and in the worst scenario, a toxic substance that can have detrimental effects on the host!

Whether FMT procedure is mature enough to be used in treating human diseases or not, there might be several concerns that should be carefully thought of. First of all, the duration and frequency of FMT therapy for each disease may differ between patients. The primary aim of FMT is to restore and reconstitute the normal microbiome of the gut in patients using specially packed stool materials from healthy individuals. Using FMT treatment for recurrent *C. difficile* (RCDI) infection as an example, Khan et al. have consolidated findings from several randomized clinical trials that inspected the efficacy of FMT (Khan et al., 2018). The analysis revealed that the FMT procedure has superior long-term efficacy as seen with prolonged disease-free (post-procedural) intervals compared to other medical treatments. Furthermore, 70–75% of patients recovered from RCDI with just a single infusion of FMT with the restoration of the microbiome (by evaluating the ratio of *Bacteroidetes* to *Firmicutes*), and the efficacy further rises to 90% when multiple FMT infusion is performed (Cammarota et al., 2015; Johnson and Gerding, 2017; Fareed et al., 2018; Khan et al., 2018). Another key point is that there was no significant difference observed in terms of delivery route/methods; regardless of in enema form or capsules containing frozen stool materials, FMT promoted recovery in patients with recurrent *C. difficile* infections (Hui et al., 2019). Then again, as the gut microbiome is a dynamic environment and can be affected by multiple factors, there is still much room to explore the frequency of FMT procedures for other diseases (e.g., ulcerative colitis, etc.) before coming up with the recommended guidelines for clinical use. A quick search on the clinical trial registry (ClinicalTrial.gov) maintained by the United States National Library of Medicine at the National Institutes of Health looking at FMT yielded a total of 242 studies. While several studies celebrated the triumph of FMT, a portion of them reported no significant improvements post-FMT treatments for diseases other than RCDI (attributed to small sample size and characteristics of recruited patients) (Goyal et al., 2016; Aroniadis et al., 2019; Saha et al., 2019; Yu et al., 2020). A randomized clinical trial conducted by Leong and the team explained a difference in microbiome at baseline between FMT donor and obese participant; donor had higher abundance of *Akkermansia muciniphila* which is known as a beneficial player in metabolism and confers the host protection against metabolic disorders including obesity (Leong et al., 2020; Xu et al., 2020). Even though there was a shift in microbiome composition among participants post-FMT (which maintained up to 12 weeks), no weight loss was observed in adolescence post-FMT treatment (Leong et al., 2020). At this point in time, more thoughts need to be placed on designing the treatment plan using FMT, including “dose” and method of delivery (oral or enema), alongside with figuring out important microbe(s) in different diseases and their mechanisms to achieve optimal long-term treatment outcome (Goyal et al., 2016; Yu et al., 2020).

Comparatively, researchers have also begun inspecting the potential use of FMT in combination with the current medical regime. As the gut microbiome acts as an important key player in immune response, the presence of pro-inflammatory microbes can trigger a vicious cycle of inflammation, breaking down the mucosal barrier and subsequently trigger widespread damage in the body system, as observed in ulcerative colitis and also neurodegenerative diseases. Thus, several clinical trials have decided to take a leap of faith, combining currently in-use chemotherapy drugs with FMT in the hope of eradicating cancer/tumor cells by modulating the immune response in the body. An ongoing Phase II clinical trial reported by Hassane Zarour and Merck Sharp & Dohme Corp., is currently investigating the potential of FMT to be used along with pembrolizumab to treat patients with melanoma (U.S. National Library of Medicine: Clinicaltrials.gov, n.d). Similarly, for infectious agents like *C. difficile* infections, it might be worthwhile to consider a combination of bacteriophage therapy with FMT rather than FMT alone to ensure higher efficacy of treatment. Some studies have described the “bottleneck” in search of bacteriophage for bacterial infections, even though bacteriophage may provide the way out in combating multidrug-resistant organisms (Hargreaves and Clokie, 2014; Letchumanan et al., 2016; Nale et al., 2016; Cisek et al., 2017). Under these circumstances, bacteriophage can act similarly like an “adjuvant” or booster along with FMT procedure to ensure the suppression of certain pathogens while reinstating the healthy “eubiosis” status of the gut.12pt

Lest we forget, we are still at war against one of the biggest threats now, severe acute respiratory syndrome coronavirus 2 (SARS-CoV-2), which causes the disease known as COVID-19 (that stands for Coronavirus disease 2019) and has claimed more than a million lives worldwide (Dong et al., 2020; Ser et al., 2020; Tan et al., 2020). After the announcement of COVID-19 as a pandemic on March 11, 2020, the FDA provided an alert update to inform clinicians and patients on the potential risk of the transmission of SARS-CoV-2 through the FMT process (World Health Organization [WHO], 2020; US Food and Drug Administration, 2020b). Concurrently, the agency also recommended that FMT product manufactured from stool donated on or after December 1, 2019, should not be used clinically until additional tests and criteria are met (e.g., donor screening for SARS-CoV-2 symptoms and diagnosis, extra confirmatory tests to detect the presence of SARS-CoV-2 virus or RNA in donated stool) (Openbiome, 2020; US Food and Drug Administration, 2020b). Although researchers are getting a better understanding of this notorious coronavirus, it is still too early to ban FMT altogether in treating human diseases in the current situation. Equally important are the tests for SARS-CoV-2 in donors and their stool, ensuring the proper handling of the specimen is one of the aspects that should be investigated to prevent cross-contamination and maintain the chain of custody (Bharat et al., 2020; Kanamori et al., 2020; Rizou et al., 2020; Ryu et al., 2020).

## Conclusion

The safety and quality check of stool material remains a vital concern for FMT treatment. With the advancement of sequencing tools, this issue may be solved in the nearest future simply by introducing a series of recommended criteria like population ratio of beneficial/protective organisms versus those with pathogenic risk and high drug resistance profile. Looking over the past, researchers are giving human stool “another chance” at contributing to human health, to a certain extent by using stool from healthy humans as a “role model” to re-educate microbial populations in patients. FMT procedure might still be far from perfect, but with the joint effort from researchers and clinicians worldwide, the FMT procedure may come as an easily accessible treatment with lower toxicity than other synthetic drugs, making it worthwhile to be explored and improved at the moment.

## Author Contributions

H-LS and VL drafted the manuscript. B-HG, SW, and L-HL contributed to the revision of the manuscript and provided vital guidance on the scientific content. H-LS, B-HG, and L-HL conceptualized the research topic. All authors read and approved the final manuscript.

## Conflict of Interest

The authors declare that the research was conducted in the absence of any commercial or financial relationships that could be construed as a potential conflict of interest.
